# Responses of sap flow, leaf gas exchange and growth of hybrid aspen to elevated atmospheric humidity under field conditions

**DOI:** 10.1093/aobpla/plu021

**Published:** 2014-05-15

**Authors:** Aigar Niglas, Priit Kupper, Arvo Tullus, Arne Sellin

**Affiliations:** 1 Institute of Ecology and Earth Sciences, University of Tartu, Lai 40, 51005 Tartu, Estonia; 2 Institute of Forestry and Rural Engineering, Estonian University of Life Sciences, Kreutzwaldi 5, 51014 Tartu, Estonia

**Keywords:** Atmospheric humidity, canopy conductance, climate change, net photosynthesis, photosynthetic capacity, relative stomatal limitation, stomatal conductance, water-use efficiency.

## Abstract

An increase in average air temperature and frequency of rain events is predicted for higher latitudes by the end of the 21st century, accompanied by a probable rise in air humidity. We currently lack knowledge on how forest trees acclimate to rising air humidity in temperate climates. We analysed the leaf gas exchange, sap flow and growth characteristics of hybrid aspen (*Populus tremula* × *P. tremuloides*) trees growing at ambient and artificially elevated air humidity in an experimental forest plantation situated in the hemiboreal vegetation zone. Humidification manipulation did not affect the photosynthetic capacity of plants, but did affect stomatal responses: trees growing at elevated air humidity had higher stomatal conductance at saturating photosynthetically active radiation (*g*_s sat_) and lower intrinsic water-use efficiency (IWUE). Reduced stomatal limitation of photosynthesis in trees grown at elevated air humidity allowed slightly higher net photosynthesis and relative current-year height increments than in trees at ambient air humidity. Tree responses suggest a mitigating effect of higher air humidity on trees under mild water stress. At the same time, trees at higher air humidity demonstrated a reduced sensitivity of IWUE to factors inducing stomatal closure and a steeper decline in canopy conductance in response to water deficit, implying higher dehydration risk. Despite the mitigating impact of increased air humidity under moderate drought, a future rise in atmospheric humidity at high latitudes may be disadvantageous for trees during weather extremes and represents a potential threat in hemiboreal forest ecosystems.

## Abstract (precis)

We demonstrate that higher air humidity mitigates the effect of low soil water availability on broadleaved trees during dry years by reducing stomatal limitation to photosynthesis, allowing higher net photosynthetic rates and supporting higher growth rates. At the same time, rising atmospheric humidity increases sensitivity of canopy conductance to water deficit and reduces the responsiveness of intrinsic water-use efficiency. The results imply that a future rise in atmospheric humidity at high latitudes may be disadvantageous in evenly rainy/humid years and expose trees to higher dehydration risk during weather extremes, although mitigating the impact of soil water deficit under moderate drought.

## Introduction

With rapid increases in global industrial development, fossil fuel use and changing land-use practices, atmospheric CO_2_ concentration ([CO_2_]) is expected to double within the 21st century. This increase will result in global climate changes: global mean water vapour concentration, evaporation and precipitation rates, as well as global mean surface temperature are projected to increase during the 21st century (IPCC 2007). These changing climate factors along with rising [CO_2_] affect the physiological performance of plants: CO_2_ assimilation, transpiration, stomatal conductance (*g*_s_) and ultimately plant growth and productivity.

The impact of the most common consequences of climate change—drought, high temperature and high atmospheric vapour pressure deficit (VPD)—on photosynthesis and water use in C_3_ plants has been quite well studied, because the occurrence of extreme temperatures, soil water deficit and high VPD, as well as their interactions, alters the physical properties and yield of plants, which are important to agriculture and forestry (Fletcher *et al.* 2007; Guha *et al.* 2010; Estrada-Campuzano *et al.* 2012; Kuster *et al.* 2013; Li *et al.* 2013; Sapeta *et al.* 2013). Considerably less is known of the effect of increasing atmospheric humidity on plants. Increases in precipitation are considered very likely at high latitudes in the long-term perspective (IPCC 2007). Precipitation is predicted to increase in northern Europe, especially in winter, and to decrease in southern and central Europe in summer (Räisänen *et al.* 2004). There might also be fewer dry days at higher latitudes by the end of the 21st century (IPCC 2007). Increasing rainfall frequency results in higher relative air humidity at local or regional scales.

The leaves of plants grown at high relative humidity (RH) have larger stomata, larger stomatal pore aperture and length, and significantly lower stomatal density due to larger epidermal cells than in plants grown at moderate RH (Torre *et al.* 2003; Nejad and Van Meeteren 2005; Arve *et al.* 2013). Therefore, decreasing VPD may lead to increased stomatal conductance and to a consequent increase in transpiration in some plant species grown at high RH (Pospíšilová 1996; Fordham *et al.* 2001; Nejad and Van Meeteren 2005). Nevertheless, most findings suggest that a decrease in VPD generally leads to decreased steady-state leaf transpiration or sap flux density in a wide range of tree species from different habitats (Pataki *et al.* 1998; Meinzer 2003; Bovard *et al.* 2005; Hölscher *et al.* 2005). Our previous studies have demonstrated decreased sap flux density in response to increased air humidity in silver birch (*Betula pendula*) and hybrid aspen (*Populus tremula* × *P. tremuloides*) trees in moist summers (Kupper *et al.* 2011).

Growing at high RH not only alters stomatal morphology, but stomatal functioning as well (Fanourakis *et al.* 2010, 2011). It is known that RH is a key environmental factor mediating changes in stomatal sensitivity to CO_2_ (Talbott *et al.* 2003). Moreover, RH affects stomatal response to water availability and drought. Plants grown at high RH are less hydrosensitive than plants grown at moderate RH: stomata of high-RH-grown leaves are less sensitive to decreases in leaf water potential than moderate-RH-grown leaves, and the homogeneity, speed and degree of stomatal closure are less in high-RH-grown plants (Nejad and Van Meeteren 2005; Rezaei Nejad *et al.* 2006; Rezaei Nejad and Van Meeteren 2008). Therefore, plants developed under moderate RH are able to retain higher water status due to more efficient stomatal control. Arve *et al.* (2013) revealed that stomata developed under high RH respond to neither darkness nor drought, but remain open. Thus, high RH may even override the signals given by darkness. The stomata of plants growing in naturally waterlogged soil are also less sensitive to decreasing VPD than those of plants growing in well-drained soil (Sellin 2001).

High RH does not change only the stomatal characteristics of plants. Our previous experiments with silver birch and hybrid aspen have shown that elevated atmospheric RH lowers leaf nutritional status by altering nutrient movement via mass flow in soil and lowering nutrient transfer through xylem flow into leaves (Tullus *et al.* 2012*a*; Sellin *et al.* 2013). The changes in leaf nutrient content and P : N ratio in turn cause a decline in photosynthetic capacity and ultimately changes in tree growth rate.

Experiments on stomatal responses to air humidity and plant stress resistance are typically carried out in greenhouses or growth chambers with seedlings or saplings growing in pots. The objective of the present study was to investigate how artificially increased RH during leaf development affects the sap flow, stomatal responses and photosynthetic parameters of hybrid aspen (*P. tremula* × *P. tremuloides*) under free-air conditions. Hybrid aspen is a fast-growing deciduous tree species suitable for short-rotation forestry in the relatively cold climate of northern Europe (Tullus *et al.* 2012*b*). Our aim was to test the following hypotheses. (i) Trees grown at higher atmospheric humidity have higher stomatal conductance and lower water-use efficiency (WUE) than control trees. (ii) The photosynthetic capacity of leaves developed in humid air is lower because of reduced nitrogen uptake due to lower transpirational flux density. (iii) Plants grown in a more humid atmosphere are unable to adjust their WUE quickly because of acclimation to lower VPD or possible stomatal malfunction.

## Methods

### Study area and sample trees

**Figure 1.**
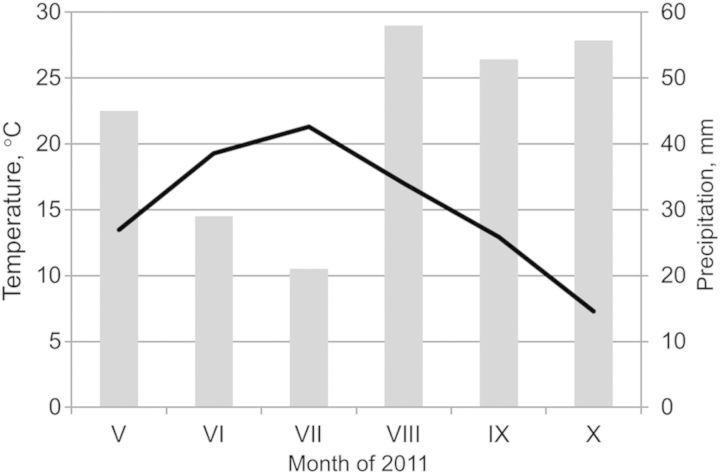
Weather data in the growing period of 2011: the dark line indicates monthly average air temperature, and the grey bars indicate monthly precipitation.

Studies were performed on hybrid aspen (*P. tremula* × *P. tremuloides*) saplings growing in an experimental forest plantation at the free-air humidity manipulation (FAHM) site, situated at Rõka village (58°24′N, 27°29′E, 40–48 m ASL) in eastern Estonia, representing a hemiboreal vegetation zone. The long-term average annual precipitation in the region is 650 mm and the average temperature is 17.0 °C in July and −6.7 °C in January. In the study year (2011) drought conditions prevailed in June and July (Fig. 1). The growing season lasts 175–180 days from mid-April to October. The soil is a fertile endogenic mollic planosol (WRB) with an A-horizon thickness of 27 cm. Total nitrogen content is 0.11–0.14 %, C/N ratio is 11.4 and pH is 5.7–6.3.

**Figure 2.**
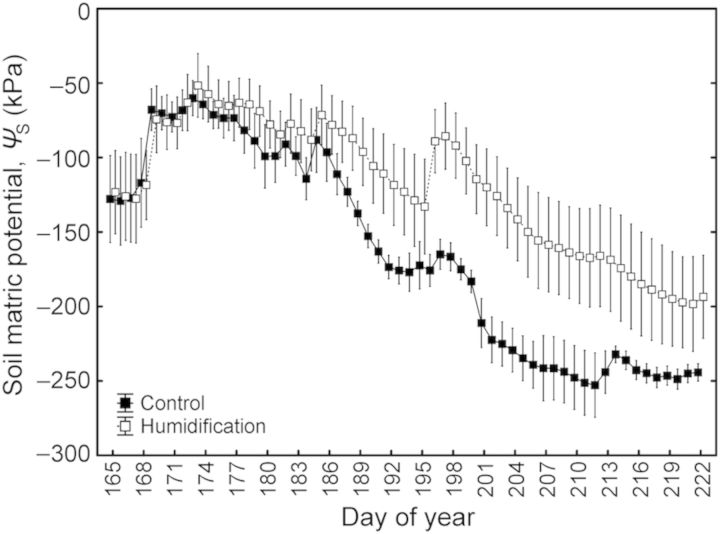
Daily average values of soil water potential at a depth of 15–30 cm in control and humidification plots from June to August in 2011. Scale bars denote SEM.

**Figure 3.**
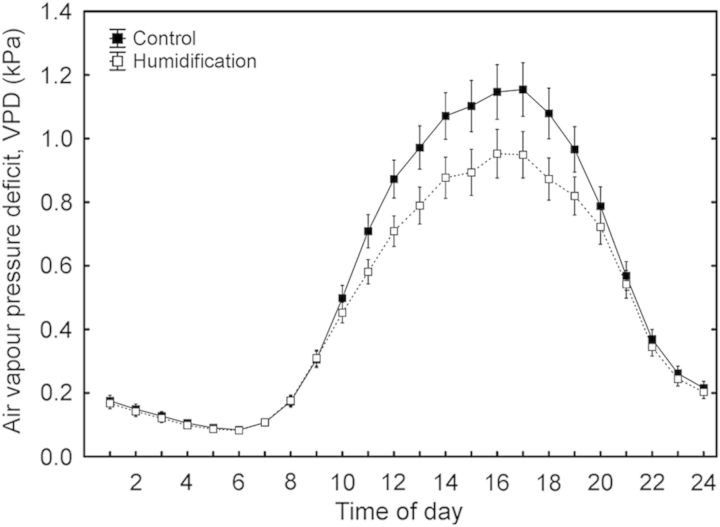
Hourly average values of air VPD in control and humidification plots from June to August in 2011. Scale bars denote SEM.

The study site, established on an abandoned agricultural field in 2006–07, is a fenced area of 2.7 ha containing nine circular experimental plots (diameter 14 m) planted with hybrid aspen and silver birch (*B. pendula*) and surrounded by a buffer zone. One-year-old micropropagated hybrid aspen plants were planted in the experimental area in the autumn of 2006. The stand density in the buffer zone is 2500 trees ha^−1^, and in the experimental plots, 10 000 trees ha^−1^. The computer-operated FAHM system, based on an integrated approach of two different technologies—a misting technique to atomize/vaporize water and FACE-like technology to mix humidified air inside the plots—enables RH of the air to increase by up to 18 % over the ambient level during the humidification treatment, depending on the wind speed inside the experimental stand. The humidification is applied during daytime 6 days a week throughout the growing period if ambient RH is <75 % and mean wind speed is <4 m s^−1^. As a long-term average, RH is 7–8 % greater in humidified plots (**H** treatment) than in control plots (**C** treatment). A detailed description of the FAHM site and technical setup is presented in Kupper *et al.* (2011). The treatment began in June 2008; sap flow and gas exchange were measured in the summer months of 2011. Soil water potential (*Ψ*_S_) was recorded at depths of 15 and 30 cm with EQ2 equitensiometers (Delta-T Devices, Burwell, UK) in eight replications per plot. The daily average *Ψ*_S_ varied from June to August and was ∼25 % higher in the humidification treatment (Fig. 2). The air temperature (*T*_a_) and RH were measured 1.5–3.5 m above the ground with 2–4 HMP45A sensors (Vaisala, Helsinki, Finland) per plot. Sensor readings were collected every 1 min and stored as 10-min average values with a data logger (DL2e; Delta-T Devices). Air VPD was calculated from *T*_a_, saturated vapour pressure and RH. The daily average VPD in the humidification treatment was 15 % lower than the control in the summer of 2011 (Fig. 3).

### Sap flow measurements

Xylem sap flow in the stems of sample trees was measured with FLOW4 sap flow systems (Dynamax Inc., Houston, TX, USA). Six trees from control plots (C1, C2, C4) and four trees from humidification plots (H1, H2) were fitted with sap flow gauges (SGB35-WS) and sampled episodically from June to August 2011. Sap flow data were recorded every 1 min and stored as 30-min averages. Sap flux density (*F*; g m^−2^ h^−1^) was calculated as sap flow divided by whole-tree foliage area estimated by mean sapwood-to-leaf-area ratios (Huber value, HV) measured in nine **C** (3.08 × 10^−4^ m^2^ m^−2^) and nine **H** (3.09 × 10^−4^ m^2^ m^−2^) trees using destructive sampling. Foliage area, measured with a LI 3100C optical area meter (LI-COR Biosciences, Lincoln, NE, USA), was on average 31 % greater in **C** trees compared with **H** trees. Whole-tree canopy conductance to water vapour (*g*_C_; mmol m^−2^ s^−1^) was computed from the data of sap flux density (mmol m^−2^ s^−1^) using a simplified Penman–Monteith equation (Komatsu *et al.* 2006; Sellin and Lubenets 2010):

(1) $$g_{C}=\frac{F\times P}{\text{VPD}},$$

where *P* is atmospheric pressure (kPa) and VPD is air vapour pressure deficit (kPa).

### Gas exchange measurements

We sampled gasometrically nine trees (mean height 3.8 m) from **C** plots and nine trees (mean height 3.3 m) from **H** plots (i.e. three trees per sample plot) for 1 month, from mid-July to mid-August. Measurements were performed on rainless misting-free days on intact fully expanded leaves *in situ* with a portable photosynthesis system LCpro+ (ADC BioScientific, Great Amwell, UK) at constant air humidity (13 mbar), CO_2_ concentration (*C*_a_ = 360 µmol mol^−1^) and temperature of the leaf chamber (25 °C). Leaf-to-air vapour pressure difference was relatively similar in the two treatments: on average 2.12 kPa for **C** plants and 1.99 kPa for **H** plants. To generate photosynthetic light response curves (*A*/*Q* curves), four leaves per tree were sampled from the middle part of the crown with an instrument equipped with an LED light source. The measurements started with photosynthetically active radiation (PAR) at 1196 µmol m^−2^ s^−1^, then decreased stepwise to 9 µmol m^−2^ s^−1^ and increased stepwise from 1196 to 1803 µmol m^−2^ s^−1^. Intrinsic water-use efficiency, expressed as the ratio of net photosynthesis (*A*_n_) to stomatal conductance to water vapour (*g*_s_), was determined at two levels of irradiance: at 400–600 µmol m^−2^ s^−1^ when IWUE was usually at a maximum (IWUE_max_) and at light intensities corresponding to full sunlight (IWUE_sat_; *Q*≥ 1400 µmol m^−2^ s^−1^).

The response of net photosynthesis to varying intercellular CO_2_ concentration (*C*_i_)—*A*/*C*_i_ curves—was also determined on intact leaves (four leaves per tree) *in situ* at constant air humidity (13 mbar), temperature of the leaf chamber (25 °C) and at saturating irradiance (1500 μmol m^−2^ s^−1^). External CO_2_ concentration (*C*_a_) was supplied in 11 steps, decreasing from 360 to 60 µmol mol^−1^ and then increasing from 450 to 1600 µmol mol^−1^. In addition to IWUE_max_ and IWUE_sat_ calculated from the data of *A*/*Q* curves, IWUE_in_ (initial IWUE) was determined using initial values of the *A*/*Q* and *A*/*C*_i_ sequences when external [CO_2_] was 360 µmol mol^−1^.

### Tree growth assessment

Tree height (*H*, cm) and stem diameter at 30-cm height (*D*, mm) of all aspen trees growing at three **C** and three **H** plots were measured before and after the 2011 growing season. *H* was measured with a telescopic Nedo mEssfix-S measuring rod (Nedo GmbH & Co.KG, Dornstetten, Germany) and stem diameter with a LIMIT digital caliper (Luna AB, Alingsås, Sweden). Current annual increment of the trees (Δ*H*, Δ*D*) was estimated as the difference between the two measurements. Relative increment (Δ*H*_rel_, Δ*D*_rel_) was expressed as the ratio of Δ*H* and Δ*D* to their respective characteristics at the beginning of the growing season. The ratio of *H*:*D* was defined as tree slenderness (*S*).

### Data analysis

Statistical data analysis was carried out using Statistica, Ver. 7.1 (StatSoft Inc., Tulsa, OK, USA). Repeated-measures analysis of variance (ANOVA) was used to compare the sap flux density (*F*) and canopy conductance to water vapour (*g*_C_) between trees from the control and the misting treatment. The daily averages of *F* and *g*_C_ were analysed altogether on 31 days from 1000 to 1700 h from 14 June to 7 August 2011 (DOY: 165–176, 197–201, 206–219). Linear regression analysis was carried out to estimate relationships between *F*, *g*_C_, VPD and *Ψ*_S_. The normality of the regression residuals was checked using the Shapiro–Wilk test.

The gasometric data were analysed with Photosyn Assistant, Ver. 1.2 software (Dundee Scientific, Dundee, UK). The *A*/*Q* curves were fitted as a non-rectangular hyperbola expressed as a quadratic equation by Prioul and Chartier (1977). The initial slope of the curve expresses the apparent quantum efficiency (*ϕ*), whereas the *X* and *Y* axes intercepts, respectively, correspond to the light compensation point (*Q*_comp_) and apparent dark respiration (*R*_d_), and the upper asymptote approximates the light-saturated rate of photosynthesis (*A*_max_). An additional parameter—convexity (*θ*)—is required to describe the rate of bending between the linear increase and the maximum value. Sub-stomatal cavity CO_2_ concentration (*C*_i_) was calculated using the model of von Caemmerer and Farquhar (1981).

The *A*/*C*_i_ curves were analysed according to the biochemical model proposed by Farquhar *et al.* (1980), and subsequently modified by Harley and Sharkey (1991) and Harley *et al.* (1992). This model enables estimation of the CO_2_ compensation point (*Γ*), the maximum rate of carboxylation by Rubisco (*V*_C max_), the PAR-saturated rate of electron transport (*J*_max_) and the rate of triose phosphate utilization (*V*_TPU_), which indicates the availability of inorganic phosphorus for the Calvin cycle (Sharkey 1985). The relative stomatal limitation on photosynthesis (*L*_S_), an estimate of the proportion of the reduction in photosynthesis attributable to CO_2_ diffusion between the atmosphere and intercellular space, was calculated from the *A*/*C*_i_ curves as follows (Farquhar and Sharkey 1982; Tissue *et al.* 2005; Huang *et al.* 2008):

(2) $$L_{S}=\left(\text{1}-\frac{A_{n}}{A_{0}}\right)\text{100,}$$

where *A*_n_ is the net photosynthetic rate at normal *C*_a_ (360 µmol mol^−1^) and *A*_0_ is the photosynthetic rate when *C*_i_ ( = 360 µmol mol^−1^) equals *C*_a_. Under these conditions, *A*_0_ is the rate of photosynthesis that would occur if there were no diffusive limitation to CO_2_ transfer through stomatal pores. The effect of humidification on gas exchange parameters was analysed by applying a nested analysis of variance with fixed factors of ‘Treatment’, ‘Experimental plot’ and ‘Soil water potential’ (a continuous variable), the second nested in the first. As plant physiological traits were more strongly related to the soil water potential measured at 30-cm depth (*Ψ*_30_), we used this parameter as an index of soil water status. Because of drought development during the measurement period, we divided the datasets of both treatments into two groups according to *Ψ*_30_ (<−204 kPa for drier soil and ≥−204 kPa for moister soil in **C** plots; <−163 and ≥163 kPa in **H** plots, respectively) and analysed gas exchange data also separately for these conditions.

Student's *t*-test was applied to estimate the treatment effect on the growth characteristics of individual trees across all experimental plots. Analysis of variance models were used to study the effects of ‘Treatment’ and ‘Experimental plot’ (nested in treatment) or ‘Treatment’ and ‘Soil water potential’ as a continuous covariate on the growth characteristics. Means and upper and lower quartiles of daily average soil water potentials (*Ψ*_S_mean_, *Ψ*_S_Q25_, *Ψ*_S_Q75_) across the growing season were used as covariates in separate models (Table 1). When exploring the variance of total and relative growth increment in 2011, tree size (*H* or *D* at the end of the previous growing season) was included as a covariate. Type IV sums of squares were used in the calculations; post hoc mean comparisons were conducted using Tukey's HSD test.

**Table 1.** Soil water potential (kPa) estimates of the experimental plots: *Ψ*_S_mean_, mean across the growing season; *Ψ*_S_Q25_, lower quartile; *Ψ*_S_Q75_, upper quartile.

Plot	*Ψ*_S_mean_	*Ψ*_S_Q25_	*Ψ*_S_Q75_
C1	−197	−240	−152
C2	−191	−217	−177
C4	−196	−217	−185
H1	−56	−76	−33
H2	−194	−221	−175
H4	−124	−151	−97

## Results

### Sap flux density and canopy conductance

**Figure 4.**
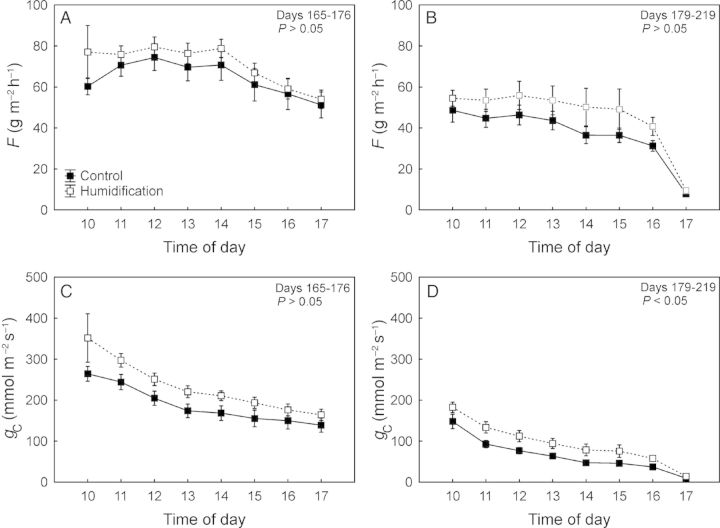
Daily average values of sap flux density (*F*) and canopy conductance to water vapour (*g*_C_) in control and humidification plots during mist fumigation from June 14 to June 25 (DOY: 165–176; A and C) and July 15 to August 7 (DOY: 197–219; B and D), 2011. Scale bars denote SEM.

**Figure 5.**
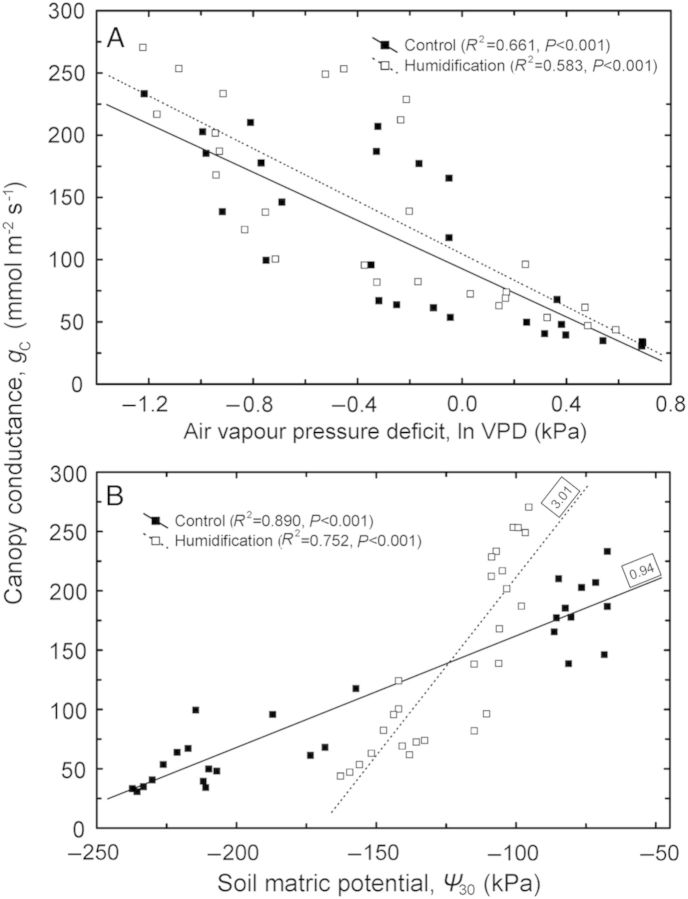
Variation in daily average canopy conductance to water vapour (*g*_C_) depending on atmospheric VPD (A) and bulk soil water potential at a depth of 30 cm (*Ψ*_30_; B). The numbers by the regression lines indicate the respective slopes.

Although canopy conductance (*g*_C_) was significantly higher (22 %; *P* < 0.05) under humidification across the whole study period, the difference between the treatments was statistically insignificant for days 165–176 (Fig. 4C) when the soil water potential did not differ between the **C** and **H** plots (Fig. 2). Also the sap flux density in the **H** treatment was on average 13 % higher than in the **C** treatment, although the difference was not significant (Fig. 4A and B). *g*_C_ decreased with increasing VPD (*P* < 0.001) in both the **C** and **H** plots; the response patterns were completely coincident and the slopes of the respective regression lines did not differ between the treatments (Fig. 5A). *g*_C_ also decreased with decreasing *Ψ*_S_ (*P* < 0.001), while the treatments demonstrated contrasting sensitivities (d*g*_C_/d*Ψ*_S_) to developing soil water deficit—the corresponding slopes were 0.94 and 3.01 for control and humidified trees, respectively (Fig. 5B).

### Leaf gas exchange

Average net photosynthesis (*A*_n_) tended to be slightly greater in trees growing at elevated atmospheric humidity than those grown at ambient RH, although the treatment means did not differ statistically throughout the experiment (Table 2). Analysis of variance revealed that the humidity treatment affected stomatal response, but not leaf photosynthetic traits (Tables 2 and 3). Specifically, there were significant differences in means of stomatal conductance to water vapour measured at saturating PAR (*g*_s__sat_) and IWUE between the treatments: *g*_s sat_ was 32 % higher and IWUE_in_ 16 % lower in the **H** treatment than in **C** trees (Table 2).

**Table 2.** Leaf gas exchange characteristics of hybrid aspen growing under control and humidification treatment. Each value is the mean ± SE; the means are compared with Tukey's test. NS, not statistically significant.

Parameter	Treatment		Significance level (*P*)
	Control±SE	Humidification±SE	
*A*_n_ (μmol m^−2^ s^−1^)	10.5±0.4	11.3±0.4	NS
*g*_s_ (mol m^−2^ s^−1^)	0.22±0.01	0.28±0.01	0.002
*g*_s sat_ (mol m^−2^ s^−1^)	0.19±0.02	0.25±0.02	0.040
IWUE_in_ (μmol mol^−1^)	53.4±1.5	45.1±1.5	<0.001
IWUE_max_ (μmol mol^−1^)	62.3±2.29	54.4±2.19	0.015
IWUE_sat_ (μmol mol^−1^)	56.8±1.88	48.81±2.19	0.008
*L*_S_ (%)	41.3±1.01	37.7±1.01	0.016
*A*_max_ (μmol m^−2^ s^−1^)	12.9±0.6	12.9±0.6	NS
*V*_C max_ (μmol m^−2^ s^−1^)	56.5±2.9	59.9±2.7	NS
*J*_max_ (μmol m^−2^ s^−1^)	173±10	196±11	NS

**Table 3.** Effects of treatment, plot and soil water potential at a depth of 30 cm (*Ψ*_30_) on gas exchange characteristics. NS, not statistically significant.

Characteristic	Factor	Significance level (*P*)
*A*_n_ (μmol m^−2^ s^−1^)	Treatment	NS
	Plot (nested in treatment)	<0.001
	*Ψ*_30_	0.013
*g*_s_ (mol m^−2^ s^−1^)	Treatment	NS
	Plot (nested in treatment)	0.002
	*Ψ*_30_	NS
*g*_s sat_ (mol m^−2^ s^−1^)	Treatment	0.027
	Plot (nested in treatment)	0.022
	*Ψ*_30_	NS
IWUE_in_ (μmol mol^−1^)	Treatment	0.013
	Plot (nested in treatment)	0.002
	*Ψ*_30_	NS
IWUE_max_ (μmol mol^−1^)	Treatment	<0.001
	Plot (nested in treatment)	0.005
	*Ψ*_30_	0.003
IWUE_sat_ (μmol mol^−1^)	Treatment	<0.001
	Plot (nested in treatment)	0.012
	*Ψ*_30_	0.007
*A*_max_	Treatment	NS
	Plot (nested in treatment)	NS
	*Ψ*_30_	NS
*V*_C max_ (μmol m^−2^ s^−1^)	Treatment	NS
	Plot (nested in treatment)	NS
	*Ψ*_30_	0.045
*J*_max_ (μmol m^−2^ s^−1^)	Treatment	NS
	Plot (nested in treatment)	NS
	*Ψ*_30_	0.007
*L*_S_ (%)	Treatment	NS
	Plot (nested in treatment)	<0.001
	*Ψ*_30_	0.015

**Figure 6.**
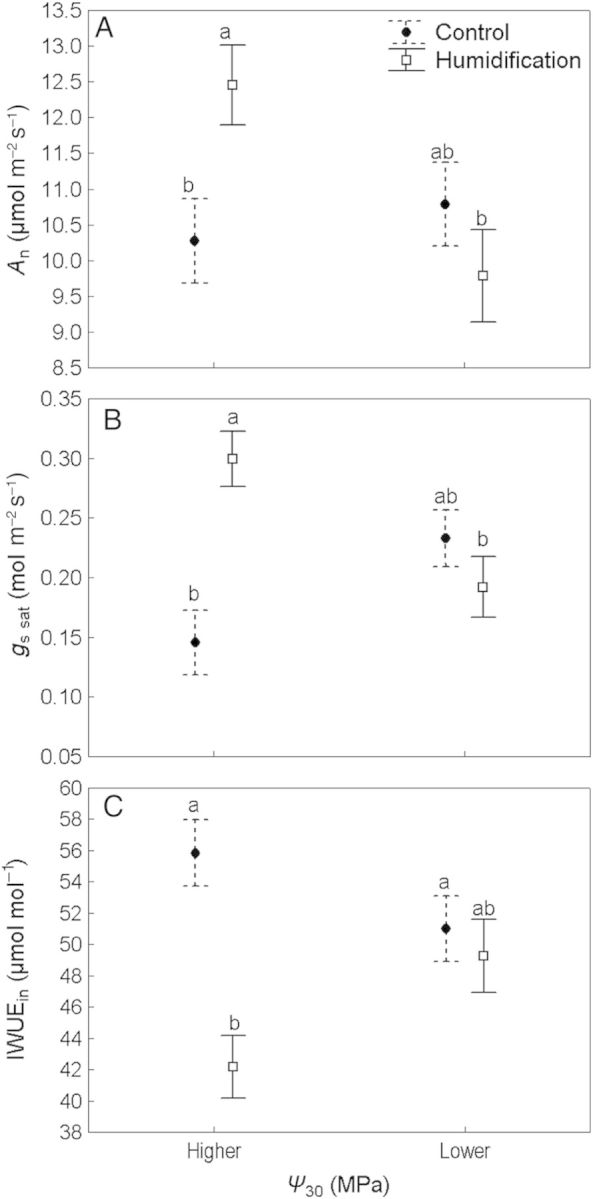
Means of net photosynthesis (*A*_n_; A), stomatal conductance to water vapour at saturating PAR (*g*_s__sat_; B) and intrinsic water-use efficiency (IWUE_in_; C) of control (closed circles) and humidified trees (open squares) depending on soil water status. Values are means ± SE; different letters denote statistically significant (*P* < 0.05) differences.

The data analysis revealed that soil water availability affected the gas exchange parameters differently within the treatments. *A*_n_ and *g*_s sat_ in the **H** treatment were significantly greater under moist soil conditions (12.45 μmol m^−2^ s^−1^ and 0.300 mol m^−2^ s^−1^, respectively) than under drier conditions (9.78 μmol m^−2^ s^−1^ and 0.192 mol m^−2^ s^−1^, respectively; Fig. 6A and B). Initial intrinsic water-use efficiency also differed with respect to soil conditions: it was lower under moist soil conditions than under drier conditions (44.2 and 49.3 μmol mol^−1^, respectively). There were no differences in *A*_n_, *g*_s sat_ and IWUE_in_ with respect to soil moisture in **C** plots (Fig. 6). It is important to notice that *g*_s_, *g*_s sat_, IWUE_in_ and *A*_max_ did not depend on *Ψ*_30_ (as a continuous variable) across the whole dataset (Table 3).

**Figure 7.**
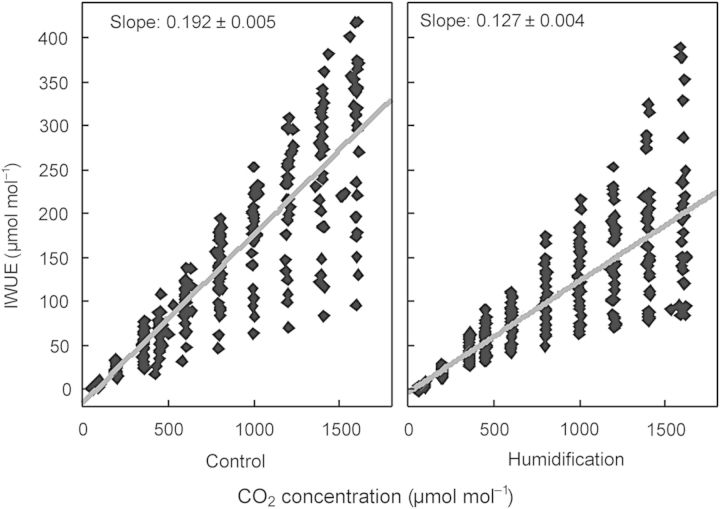
Photosynthetic WUE versus external CO_2_ concentration in control and humidification treatment.

Photosynthesis was strongly associated with *g*_s_ in both treatments: *R*^2^ = 0.84, *P* < 0.001 in **C** plots and *R*^2^ = 0.79, *P* < 0.001 in **H** plots. There was an inverse linear relationship between IWUE_in_ and *C*_i_/*C*_a_ (*R*^2^ = 0.73, *P* < 0.001), while the slopes of the corresponding regressions did not differ between the treatments (*P*> 0.05). *A*_n_ increased with rising CO_2_ concentration (*C*_a_), with significantly (*P* < 0.001) steeper response in the **H** treatment (*β* = 53.5; *R*^2^ = 0.82, *P* < 0.001) than in the control (*β* = 45.5; *R*^2^ = 0.74, *P* < 0.001). There were no differences in the *A*_n_ = *f*(*C*_i_) slopes between the treatments. *g*_s_ decreased with increasing *C*_a_, but the responses did not differ between the treatments. As a consequence, IWUE_in_ rose with *C*_a_; the slope for control trees was greater than that for humidified trees (*P* < 0.001; Fig. 7).

**Figure 8.**
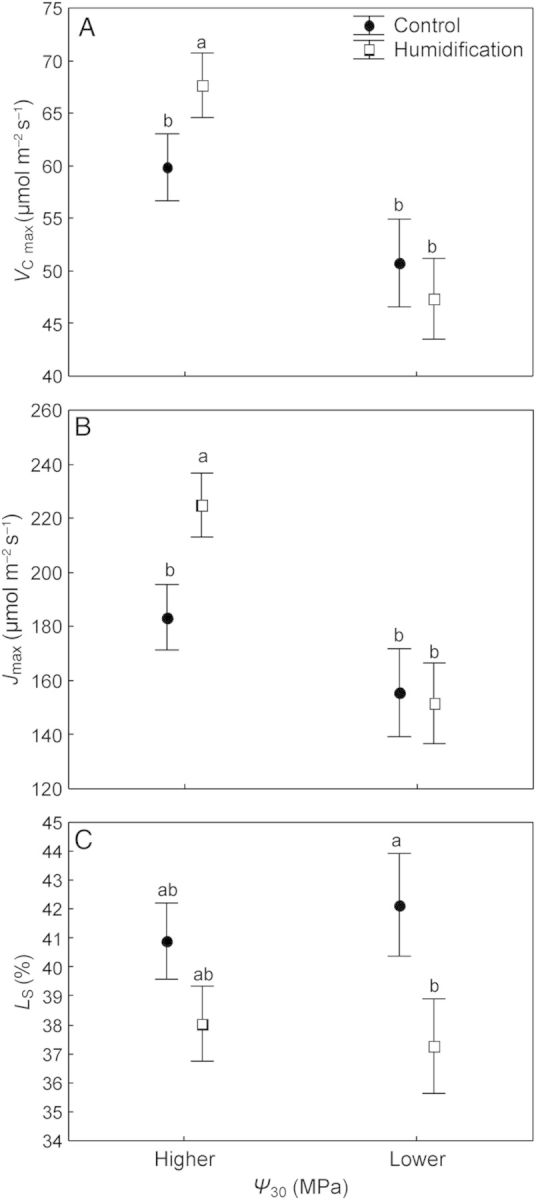
Means of maximum rate of carboxylation by Rubisco (*V*_C max_; A), PAR-saturated rate of electron transport (*J*_max_; B) and relative stomatal limitation to photosynthesis (*L*_S_; C) of control (closed circles) and humidified trees (open squares) depending on soil water status. Values are means ± SE; different letters denote statistically significant (*P* < 0.05) differences.

The maximum rate of carboxylation by Rubisco (*V*_C max_) and the maximum rate of electron transport (*J*_max_) did not differ between the treatments across the whole dataset; ANOVA revealed only an effect of soil water status on these parameters (Table 3). When the data were analysed separately in two groups (moist versus dry soil conditions), significant differences between the means of *V*_C max_ and *J*_max_ became evident only for the humidification treatment—both parameters were higher (*P* < 0.001 for both parameters) in moist soil. No variation with the soil conditions was detected in *V*_C max_ and *J*_max_ in the control trees (Fig. 8A and B). Regression analysis revealed a positive relationship between *V*_C max_ (*R*^2^ = 0.168, *P* < 0.001) and *J*_max_ (*R*^2^ = 0.151, *P* < 0.01) and *Ψ*_30_, as well as between *V*_C max_ and *J*_max_ (*R*^2^ = 0.85, *P* < 0.001) across both treatments.

The mean values of relative stomatal limitation of photosynthesis (*L*_S_) were lower in trees grown at elevated RH than in **C** trees—37.9 and 41.3 %, respectively (*P* < 0.05; Table 2), although ANOVA did not establish any significant effect of the treatment (Table 3). Net photosynthesis was negatively correlated with *L*_S_ in control trees (*R*^2^ = 0.15, *P* = 0.03), but the relationship lacked in the humidification treatment (*P* = 0.23). We found no differences in *L*_S_ with respect to soil water status in any treatment separately (Fig. 8C).

### Impact on growth rate

Saplings of hybrid aspen growing in **H** plots were significantly shorter and had narrower stems (Table 4), regardless of whether sample plot or soil water potential was included as confounding factors in the models (Table 5). The absolute and relative growth increments in 2011 were either unaffected by treatment or significantly greater in **H** plots (Tables 4 and 5). This was more pronounced when *Ψ*_S_mean_ or *Ψ*_S_Q25_ was used as a covariate in ANOVA models, although using *Ψ*_S_Q25_ yielded slightly better approximations than the two other soil water potential estimates (Table 5). Slenderness (*S*) was unaffected by treatment, but varied significantly among the experimental plots.

**Table 4.** Comparison (*t*-test) of mean (±SE) growth parameters of individual trees growing in humidified (**H**) and control (**C**) plots. NS, not statistically significant.

Growth characteristic	**C**	**H**	*t*-stat	*P*
Height (cm)	454±5.8	428±5.8	3.06	0.002
Diameter of stem at 30-cm height (mm)	32.6±0.7	29.5±0.6	3.39	<0.001
Height increment of the current year (cm year^−1^)	111±2.3	117±2.7	−1.70	NS
Relative height increment of the current year	0.34±0.01	0.39±0.01	−3.46	<0.001
Diameter increment of the current year (mm year^−1^)	8.9±0.3	8.5±0.2	1.14	NS
Relative diameter increment of the current year	0.38±0.01	0.41±0.01	−1.65	NS
Slenderness (height-to-diameter ratio)	14.4±0.2	14.8±0.2	−1.97	0.051

**Table 5.** Results from ANOVA models describing the effect of tree size (i.e. the value of the respective parameter before the start of the growing season, *C_t_*_−1_), treatment (*T*), plot and soil water potential (*Ψ*_S_mean_, *Ψ*_S_Q25_, *Ψ*_S_Q75_) on the growth parameters.

Factors		Response variables						
		*H*	*D*	Δ*H*	Δ*H*_rel_	Δ*D*	Δ*D*_rel_	*S*
Model 1								
* C_t_*_−1_	*P*	–	–	0.824	<0.001	<0.001	0.766	–
* T*	*P*	<0.001	<0.001	0.074	0.247	0.069	0.086	0.051
* *Plot (*T*)	*P*	<0.001	<0.001	<0.001	<0.001	<0.001	<0.001	0.015
	Adj. *R*^2^	0.29	0.13	0.09	0.37	0.37	0.16	0.03
Model 2								
* C_t_*_−1_	*P*	–	–	0.802	<0.001	<0.001	0.006	–
* T*	*P*	<0.001	<0.001	0.019	0.013	0.041	0.017	0.668
* Ψ*_S_mean_	*P*	<0.001	0.157	<0.001	<0.001	<0.001	<0.001	0.005
	Adj. *R*^2^	0.08	0.03	0.09	0.36	0.32	0.09	0.03
Model 3								
* C_t_*_−1_	*P*	–	–	0.880	<0.001	<0.001	0.004	–
* T*	*P*	<0.001	<0.001	0.013	<0.001	0.049	0.018	0.800
* Ψ*_S_Q25_	*P*	<0.001	0.081	<0.001	<0.001	<0.001	<0.001	0.010
	Adj. *R*^2^	0.09	0.04	0.10	0.37	0.32	0.09	0.03
Model 4								
* C_t_*_−1_	*P*	–	–	0.684	<0.001	<0.001	0.008	–
* T*	*P*	<0.001	0.002	0.083	0.077	0.075	0.052	0.622
* Ψ*_S_Q75_	*P*	<0.001	0.351	<0.001	<0.001	<0.001	<0.001	0.002
	Adj. *R*^2^	0.07	0.03	0.07	0.35	0. 32	0.08	0.04

## Discussion

### Effects on sap flow and gas exchange

The sap flux density in hybrid aspen trees changed considerably compared with that at the same experimental site in previous summers (Kupper *et al.* 2011; Tullus *et al.* 2012*a*). *F* in control plots was significantly greater than that in the humidification treatment during the rainy summer of 2009 (Kupper *et al.* 2011). The same tendency (not significant) was observed in the drier summer of 2010 (Tullus *et al.* 2012*a*). However, our current results demonstrate higher (although not statistically significant) sap flux densities in trees growing in the humidification plots (Fig. 4). The canopy conductance to water vapour in the **H** treatment was greater (*P* < 0.05) across the whole study period. This discrepancy is attributable to relatively low soil water potential in the control treatment owing to the very dry summer: *Ψ*_S_ did not rise over −80 kPa in July. The total precipitation during the 2011 growing season (May–October) was 261 mm (Fig. 1), 42 % less than the average of the three previous years (452 mm). As the air humidity manipulation did not affect sapwood-to-leaf-area ratio (HV), the ∼30 % greater leaf area of control trees was responsible for greater transpirational water losses causing faster depletion of soil water reserves and a greater decline in *Ψ*_S_ despite lower overall sap flux densities in **C** plots (Fig. 4A and B).

The response of canopy conductance to changes in VPD did not vary between the treatments, suggesting that stomatal sensitivity to atmospheric evaporative demand was not affected by the experimental manipulation. In contrast to that, *g*_C_ decreased much faster in response to falling *Ψ*_S_ in the **H** treatment than in the control (Fig. 5). The differential response of *g*_C_ to decreasing soil water availability is probably mediated by plant hydraulic conductance (Cohen and Naor 2002; Domec *et al.* 2009). Hydraulic measurements performed on aspen trees in 2010 revealed that both soil-to-branch and leaf hydraulic conductances expressed per unit leaf area were smaller in humidified trees, although growing in moister soil (A. Sellin, unpubl. res.). Under conditions of soil water deficit the lower hydraulic capacity probably becomes a crucial factor for **H** trees, limiting leaf water supply and inducing a steep decline in canopy conductance. Furthermore, an experiment with silver birch revealed that a rapid water deficit in **H** plants led to a faster decrease in hydraulic conductance—responsible for liquid water supply—compared with the decrease in *g*_s_, which limits water losses, and exposed plants to a greater risk of dehydration (Sellin *et al.* 2014).

Lowering *A*_n_ and *g*_s_ and increasing IWUE are typical responses to water stress in plant species with a drought avoidance strategy. When plants encounter a soil water deficit, abscisic acid (ABA) is synthesized in the roots and translocated to the leaf through the transpiration stream (Assmann and Shimazaki 1999); higher concentrations of ABA in leaves drive mechanisms leading to a decrease in *g*_s_ and an increase in WUE (Liu *et al.* 2005). Pantin *et al.* (2013) propose that ABA promotes stomatal closure in two ways—via its widely known biochemical effect on guard cells and via an indirect hydraulic effect through a decrease in leaf hydraulic conductance. Maintaining stable gas exchange attributes during drought development means that a plant either possesses a drought tolerance strategy or lacks adaptations with respect to drought. *Populus tremula*, one of the parent species, is known to act as a drought avoider (Possen *et al.* 2011). In our case, the lack of variation in gas exchange characteristics in **C** plots with respect to soil water status (Fig. 6) and modification of gas exchange in **H** plots by altering water availability can be explained by the lower *Ψ*_S_ values in the **C** treatment (i.e. long-term effects mediated by ABA).

Regardless of the difference in *Ψ*_S_ between the treatments, there was still an effect of the humidity manipulation on *g*_s sat_ and IWUE (Table 3). In fact, the differences in leaf gas exchange between the **C** and **H** plots are attributable to the combined effects of soil water availability and increased atmospheric humidity. As such, our first hypothesis is supported by the experiment: growing at higher RH increases stomatal conductance in trees while lowering photosynthetic WUE (Table 2), while the effect is largely mediated by changes in soil water status.

Soil water potential influenced both *V*_C max_ and *J*_max_ in hybrid aspen (Table 3). Grassi *et al.* (2005) found a positive relationship between *V*_C max_ and *Ψ*_S_ in oak and ash trees during summer, as in hybrid aspen in this study. A simulation by Grassi and Magnani (2005) indicated that 30–40 % of the biochemical limitation could be attributed to a reduction in leaf nitrogen content during droughty summers. Plant photosynthetic capacity and leaf N content expressed per leaf area are positively correlated (Grassi *et al.* 2005; van de Weg *et al.* 2012). Previous work at the FAHM experimental site has shown that rising RH reduces the water flux in silver birch (Kupper *et al.* 2011) and alters the nutritional status of leaves, leading to a decline in photosynthetic capacity (Sellin *et al.* 2013). In this experiment, humidification increased rather than decreased water flux through the trees (Fig. 4), which explains why the biochemical capacity of photosynthesis was unaffected by the manipulation, but by changes in *Ψ*_S_ (Table 3). The second hypothesis concerning reduced photosynthetic capacity of leaves due to the expectedly smaller N uptake in humidified trees remained unconfirmed. The absence of an impact of air humidity manipulation on photosynthetic machinery of hybrid aspen was also supported by chlorophyll fluorescence measurements performed in the droughty summer of 2012 (A. Niglas, unpubl. res.).

High RH can affect stomatal sensitivity by changing stomatal morphology: plants grown at higher RH have larger stomata that close to a lesser extent when leaves dry (Giday *et al.* 2013). In addition, long-term acclimation to high RH during growth increases heterogeneity in stomatal response characteristics to short-term exposure to stomatal closure-inducing factors (Nejad and Van Meeteren 2005). Experiments showing differences in stomatal sensitivity and morphology between plants grown at high and low RH have been carried out under stable/controlled environmental conditions. The conditions before a leaf is fully expanded are important determinants on whether stomatal closure capacity is affected by leaf dehydration and RH. Moreover, the degree of stomatal adaptation in expanding leaves depends on the duration and timing of the exposure to high RH (Fanourakis *et al.* 2011). The present study was performed under field conditions with natural diurnal fluctuations of RH; misting was applied when ambient RH was <75 % and could be increased to as much as 18 % (versus 60 and 95 % of RH in Fanourakis *et al.* 2011). Our data suggest that stomatal sensitivity to atmospheric VPD remained unaffected in saplings of hybrid aspen. Although we did not explore stomatal dimensions, we presume that differences in morphology and the putative morphological effect on stomatal sensitivity were rather minor as our trees grew *in natura*, in both diurnally and seasonally variable environments, under conditions requiring flexible stomatal adjustment.

The findings that stomatal conductance decreases and photosynthesis increases with rising external CO_2_ level are well-known phenomena (reviewed by Araújo *et al.* 2011). A steeper *A*_n_ response to *C*_a_ in **H** plots is attributable to higher stomatal conductance (evidenced by *g*_s max_) due to leaf development under lower VPD (Table 2). This is indirectly confirmed also by the negative correlation between *A*_n_ and *L*_S_ in the **C** treatment.

Initial intrinsic water-use efficiency responded more sensitively to *C*_a_ in **C** plots than in **H** plots (Fig. 7), testifying once more to the effect of elevated atmospheric humidity on leaf gas exchange. Because high IWUE is advantageous to plants under drought conditions, a slower response in high-RH-grown trees to changing ambient conditions may be disadvantageous in the case of abrupt climatic fluctuations becoming more frequent in the future (Easterling *et al.* 2000); these plants are not able to adjust their water use as quickly as plants grown in drier air and experience greater water loss. Thus, our results support the third hypothesis on the capacity of plants to modify WUE under changing environmental conditions, albeit not directly tested with respect to air humidity.

### Consequences on tree growth

The above-ground growth response of aspen trees to humidification in 2011 demonstrated some trends in contrast to those observed in previous years (Tullus *et al.* 2012*a*). However, the positive effect of humidification was detectable only in current-year height increments, while overall dimensions remained smaller in **H** plots, where hybrid aspen trees had grown slower than in **C** plots in the two previous experimental years (Tullus *et al.* 2012*a*). The inverse growth response is also attributable to dry weather conditions prevailing in summer 2011. Generally *Ψ*_S_ or experimental plot was the more significant factor influencing tree growth response than humidity manipulation. One must take into account that the two factors—*Ψ*_S_ and treatment—are partly interrelated, as transpirational flux through trees was lower in **H** plots (Fig. 4A; see also Kupper *et al.* 2011 and Tullus *et al.* 2012*a*) and more water was retained in the soil (Fig. 2). However, the humidity manipulation also had an impact on growth when considering the effect of *Ψ*_S_; thus, the humidification effect on tree growth was clearly not due solely to altered soil water availability. In average or rainy years, when soil water does not limit growth, lowered transpiration hinders nutrient uptake by trees in **H** plots (Tullus *et al.* 2012*a*), especially for nutrients migrating to the roots with mass flow in soil. Under these conditions increased atmospheric humidity does not improve the growth rate of hybrid aspen. Sellin *et al.* (2013) also showed that humidification treatment lowers the photosynthetic capacity and growth rate of silver birch in moist summers. In dry years, when soil water availability limits growth, the impact of this mechanism is obviously irrelevant.

## Conclusions

The current study demonstrates that higher air humidity mitigates the effect of low soil water availability on broadleaved trees during dry years by reducing stomatal limitation to photosynthesis, allowing higher net photosynthetic rates and supporting higher growth rates (relative height growth). At the same time, higher RH increases the sensitivity of canopy conductance to water deficit and reduces the responsiveness of IWUE to factors inducing stomatal closure. The present and our earlier results (Tullus *et al.* 2012*a*; Sellin *et al.* 2013, 2014) imply that a future rise in atmospheric humidity at high latitudes may be disadvantageous in evenly rainy/humid years and expose trees to a higher dehydration risk during weather extremes, although mitigating the impact of soil water deficit under moderate drought.

## Sources of Funding

This study was supported by the Estonian Science Foundation (Grant no. 8333), by the Estonian Ministry of Education and Research (target financing project SF0180025s12), and by the EU through the European Social Fund (Mobilitas postdoctoral grant MJD 257) and the European Regional Development Fund (Project No. 3.2.0802.11-0043 ‘BioAtmos’ and Centre of Excellence in Environmental Adaptation).

## Contributions by the Authors

A.N. and A.S. designed and performed the experiment, and wrote the manuscript. P.K. and A.T. performed the experiment, analysed the data and revised the paper. All authors read and approved the final manuscript.

## Conflicts of Interest Statement

None declared.
